# Detection of poliovirus by ICC/qPCR in concentrated water samples has greater sensitivity and is less costly using BGM cells in suspension as compared to monolayers

**DOI:** 10.1186/1743-422X-7-282

**Published:** 2010-10-25

**Authors:** Helene B Balkin, Aaron B Margolin

**Affiliations:** 1 Molecular, Cellular and Biomedical Sciences Department, University of New Hampshire, Durham NH USA

## Abstract

The integrated cell culture quantitative reverse transcriptase PCR (ICC/qRT-PCR) method is used in our lab to detect enteroviruses in environmental waters. Typically we utilize monolayers of 3 cell lines; buffalo green monkey kidney (BGM), human colonic carcinoma (CACO-2) and African rhesus monkey kidney (MA104) with the intent of providing one or more permissive hosts to a wide range of enteroviruses. In this study the BGM cell line was used to compare poliovirus infectivity in conventional monolayer cultures to BGM cells in suspensions. Propagated virus was subsequently amplified by qRT-PCR. Our PCR data showed lower cycle threshold (Ct) values in the suspensions which corresponded to a higher rate of infectivity than that observed in the monolayers. The difference in Ct values was determined statistically significant by One-way ANOVA (0.000). Infecting BGM cells in suspensions required less hands-on time, less chance of contamination and was more cost effective than utilizing the conventional monolayer technique.

## Findings

Viral infection is suspected in 50% of all acute gastrointestinal illness [1] with the public being at greatest risk acquiring infection through wastewaters that contaminate drinking water sources, recreational waters and shellfish harvesting waters [2].

ICC/qRT-PCR is a proven method for the rapid detection of infective enteroviruses in environmental waters [3,4]. With this technique viruses, which are generally present in low numbers, are propagated in monolayers of a host cell line which increases the PCR target. Little published research is available using cell culture systems other than monolayers to screen environmental samples [5,6]. One study reported the development of a BGM shaker culture where the cells were adapted to a suspension culture by serial passaging and using special medium and a gyratory shaker. Infectivity was compared between the adapted cells and BGM monolayers by inoculating with poliovirus 1, 2 and 3 (as well as other viruses). The suspensions showed higher log _10 _plaque forming units per mL (PFU/mL) than the monolayers [6]. In another (clinical) study, cells were infected with herpes simplex virus (HSV) in what was described as a simultaneous seeding and infection (suspension-infection) method which yielded a mean time to diagnosis of 1 day. This method became routinely used in the authors' laboratory because of its ease, sensitivity and timeliness [7]. Here we describe a comparable suspension-infection technique for detecting viruses in environmental samples that doesn't involve adapting and maintaining cells in suspension or the manipulations and procedural steps associated with conventional monolayer cell culture.

For this study the BGM cell line was chosen to demonstrate proof of concept due to its high susceptibility to enteroviruses in water samples [5,6,8] and the concomitant use of poliovirus as a standard experimental model. In addition enumeration of poliovirus in BGM monolayers is easily accomplished via neutral red plaque assay.

Three experiments were performed using in house BGM cells at passage number 94. In each trial cells were seeded into six Corning T150 cm^2 ^culture flasks with growth medium containing 43% Lebowitz L-15 modified medium (Sigma), 27% Eagle's Minimal Essential Medium (MEM), 24% HEPES (Fisher), 4% sodium bicarbonate (Sigma), 2% (w/v) L-glutamine (Sigma), 1% non-essential amino acids, 1% antibiotic/antimycotic (Cellgro), 1% kanamycin sulfate (Cellgro) and heat treated 5% (v/v) fetal bovine serum (FBS) (JRH Biosciences). The cells were incubated at 37°C in a closed system until confluent monolayers of ~ 1.5 × 10^7 ^total cells were observed. All of the monolayers were washed three times with phosphate buffered saline (PBS) (Sigma) prior to manipulation. Three of the monolayers were detached with 10 mL of trypsin EDTA (Cellgro) and transferred to corresponding 50 mL polypropylene (pp) conical tubes (Sarstedt). MEM supplemented with 2% FBS was added to each tube for a volume of 34 mL. The monolayers and suspensions were immediately inoculated with a mock sample which was prepared by dissolving 10% beef extract (BE) (Becton Dickinson) in 4 liters of deionized (DI) water at neutral pH. When the BE was thoroughly suspended the sample was concentrated by organic flocculation [9] for a final volume of 20 mL. Each sample was inoculated with 1% of 100X antibiotic/antimycotic and 0.1% of 50 ug per mL of gentamicin sulfate and incubated at 37°C for 2 hours. Post incubation the samples were stored at -80°C.

Prior to spiking the concentrated samples were quickly thawed at 37°C. They were combined for a total volume of 200 mL and then spiked with 8.5 × 10^6 ^PFU/mL poliovirus type 1 strain LSc-1 (PV 1) which was enumerated by a neutral red plaque assay. Six mL of the sample which contained 10 PFU PV1 was added to each of the three monolayers and three suspensions. The monolayers were incubated at 37°C for 80 min to allow for adsorption of the PV1. They were subsequently returned to the safety hood for the addition of MEM supplemented with 2% FBS and then returned to the incubator. The suspensions were gently swirled and the tubes were placed horizontally between Styrofoam strips with the capped end slightly elevated in a 37°C incubator.

All of the controls for the monolayers and suspensions were prepared in triplicate. Negative controls consisted of MEM supplemented with 2% FBS, unspiked concentrated sample, and PBS. The positive controls were inoculated with 100 PFU of poliovirus with the Time = 0 hour (T = 0) control being immediately frozen at -80°C.

No manual counts were performed on the monolayers or suspensions immediately before or after inoculation. Prior research by Hoyt *et al*. [10] demonstrated that it took 24-48 h for 100,000 BGM cells to double in density, therefore, no appreciable increase in number would occur. Future work using cells with densities less well characterized will be counted just prior and post inoculation.

The monolayers and suspensions were observed the following day by inverted phase contrast microscopy. As expected the monolayers were unaffected, where as in the tubes, cellular debris and both attached cells and suspended cells were seen. By day 3 post infection, partial monolayers in the flasks were observed along with rounded up and floating cells. In the pp tubes, a layer of cells was still attached and many floating cells and clumps of cellular debris were observed. On day 6 the flasks had mostly floating cells with some attached cells remaining. The tubes showed mostly CPE with few attached cells. All flasks and tubes were frozen on day 6 at -80°C and stored until the RNA extraction procedure was performed. Because of the highly lytic nature of poliovirus type 1 strain LSc-1 and the duration of the experiment only 1 freeze/thaw was performed. However future work utilizing different cell lines and viruses may require more freeze/thaw cycles.

The samples were rapidly thawed prior to nucleic acid extraction using Qiagen's QIAamp DNA mini blood columns with the following changes: the volume of ethanol was increased from 200 uL to 230 uL and the elution Buffer AE was decreased from 200 uL to 60 uL. These columns are used in our lab to extract and co-purify DNA and RNA viruses from cell lysates. One mL aliquots were spun in microcentrifuge tubes to pellet cellular debris. Two hundred microliters of the supernatant was processed and the nucleic acid was stored at -20°C.

qRT-PCR was performed on the Applied Biosystems (AB) 7300 real-time machine using the TaqMan One Step RT-PCR kit (AB). Each reaction contained 5 uL of RNA template and a panenterovirus set of primers and probe (AB). The primers and probe targeted the highly conserved 5' untranslated region of the genome [11]. The 5' and 3' end of the probe were labeled with reporter 6-carboxyfluorescein (FAM) and quencher 6-carboxytetramethylrodamine (TAMRA) respectively as depicted in Table 1. Serial 10 fold dilutions of stock poliovirus 1 LSc-1 of 8.5 × 10^6 ^PFU/mL exhibited a detection limit of 0.425 PFU (data not shown).

**Table 1:** Panentovirus primers and probe set Amplicon size and target

Forward primer	5'-CCTCCGGCCCCTGAATG-3'	197-bp highly conserved
		5'untranslated region
Reverse primer	5'-ACCGGATGGCCAATCCAA-3'	

Probe	5'-6FAM-TACTTTGGGTGTCCGTGTTTC-TAMRA-3'	

All controls and experimental groups were run in triplicate (neg, pos, and T = 0 hour not shown). The thermal profile was 48°C for 45 min, 95°C for 10 min, 45 cycles of 94°C for 15 sec and 55°C for 1 min (AB).

**Figure 1:**
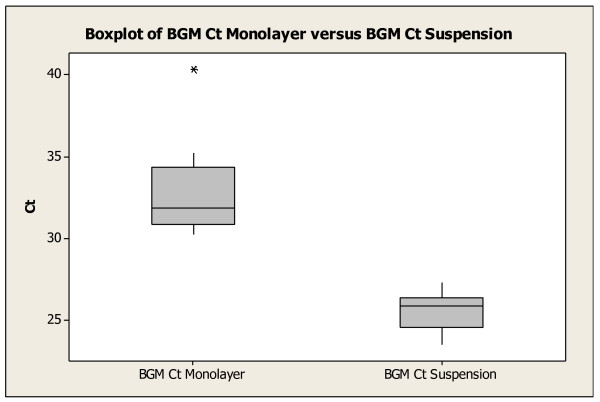
**Comparison of ICC/q RT PCR cycle threshold (Ct) values of BGM monolayers infected with 10 PFU of poliovirus type 1 (strain LSc-1) to similarly infected BGM suspensions in 50 mL pp tubes on day 6**. Each boxplot represents 3 trials run in triplicate. The average Ct values were 32.17 and 25.51 for the monolayers and suspensions respectively. One-way ANOVA was applied to interpret the Ct data. The groups were determined to be statistically significant different (p 0.000).

A boxplot was constructed from the Ct data (see Figure 1).

The ICC-qRT-PCR method allows low virus concentrations to be propagated to increase target nucleic acid. Traditional cell culture methods employ only the monolayer 2D arrangement which in this study showed lower levels of infection compared to the cells in a suspension or 3D configuration. To clarify, this was not a true suspension culture in that there was no special medium and no mechanical means to stop the cells from attaching. Upon inoculation however, the cells were in a 3D form enveloped in sample and medium. By placing the tubes horizontally the cells were prevented from pooling at the bottom and instead remained mostly in suspension with some attached to the sides. It was demonstrated in our lab (unpublished) that BGM cells in suspension on day 6 are viable. An aliquot of suspension was seeded into a flask where a monolayer growth pattern was formed.

Research by Goldstein *et al*. proved that cells in a true suspension or 3d configuration aids in poliovirus (and other viruses) infection. In a clinical setting Luker *et al*. demonstrated that even in low numbers virus infection in a suspension-infection method is detected sooner than the monolayer method and that it was not imperative that cells remain in suspension for infection to progress. Similarly, our study displayed higher PV1 infection in cells that were in suspension compared to the monolayer conformation. Trypsinization immediately before the suspensions were inoculated may have increased yield however Goldstein *et al*. did not add trypsin to the suspensions which showed a higher rate of infection.

The benefits of using the tubes were the lower risk of contamination, less manipulation required, and the vast cost difference between culture flasks and pp tubes.

In the future we expect to study adenovirus 40, 41, astrovirus, and rotaviruses which are also found in environmental waters and compare monolayers and suspensions by ICC/qPCR, and qRT-PCR

## Competing interests

The authors declare that they have no competing interests.

## Authors' contributions

HBB carried out the laboratory experiments, interpreted the results and wrote the manuscript. ABM co-interpreted the results and both authors read and approved the final manuscript.
